# Sexual Minority Status and Psychological Risk for Suicide Attempt: A Serial Multiple Mediation Model of Social Support and Emotion Regulation

**DOI:** 10.3389/fpsyt.2020.00385

**Published:** 2020-05-13

**Authors:** Cindy J. Chang, Kara Binder Fehling, Edward A. Selby

**Affiliations:** ^1^Graduate School of Applied and Professional Psychology, Rutgers University, Piscataway, NJ, United States; ^2^Department of Psychology, Rutgers University, Piscataway, NJ, United States

**Keywords:** sexual minority, suicide, social support, emotion dysregulation, lesbian, gay, bisexual

## Abstract

The current study examined the relation between sexual minority status, social support, emotion dysregulation, and suicide attempt in a community sample. A total of 388 community and college adults completed a one-time survey examining self-injury and suicidality. Findings demonstrated that that social support and emotion regulation, independently and in sequence, mediated the relation between sexual minority status and suicide attempt. The reverse mediation model with emotion regulation as the first mediator and social support as the second mediator was also significant. Social support and emotion regulation may both be related and may explain the relation between sexual minority status and suicide attempt. If replicated longitudinally, these findings shed light on specific risk factors and their interrelations, which may have important implications for preventing suicide in sexual minorities.

## Introduction

Across the U.S. population, suicide is the tenth leading cause of death, killing more than 47,000 people in 1 year (1). It has been well-established that sexual minority individuals are at especially high risk for suicidal ideation and behavior as compared to their heterosexual counterparts [e.g., (2–6)]. One meta-analysis found that lesbian, gay, and bisexual (LGB) individuals are over two times more likely to have attempted suicide in their lives compared to heterosexual individuals (5). In a community-based survey of LGB individuals, approximately one in five sexual minority adults were found to have attempted suicide (7). The increased likelihood of sexual minority individuals to attempt suicide is still observed even after controlling for presence of mental disorders (2). Research in this area consistently demonstrates that sexual minority individuals in the United States experience unique risk for suicidal behavior. However, the specific links between sexual minority status and suicide outcomes remain unclear.

Several theoretical models have attempted to explain disparities in negative mental health outcomes such as suicidal behavior. One such model is Minority Stress Theory, which posits that stressors unique to sexual minority individuals may help to explain elevated mental health disparities (8, 9). Existing research has illuminated possible minority stress processes that may contribute to suicidal ideation or behavior (10, 11). One minority stressor relevant to suicide is degree of social support. The widely researched Interpersonal Theory of Suicide (IPT) posits that suicide risk is best attributed to three main components: perceived burdensomeness, low sense of belongingness, and acquired ability to enact lethal self-injury (12). This theory takes an interpersonal approach toward understanding suicide and suggests that social alienation, or lack of social support, confers greater risk for suicide. With regards to sexual minorities, there is ample support for the role of social support and relational constructs. One study examining minority stress theory and IPT found that thwarted belongingness and perceived burdensomeness both explain suicide risk in sexual minority adults in Bavaria (13). Conversely, social support and social connectedness may serve as a protective factor against suicide (14, 15). In particular, social support has been found to mediate the relation between sexual orientation and treatment for mental disorders (16). Taken together, social support, or lack thereof, may be a stressor or resilience factor unique to sexual minorities that may help explain disparities in suicidality.

A growing body of research has also focused on psychological risk factors that may explain suicide risk disparities in sexual minorities. Hatzenbuehler (17) suggested that minority stressors increase a sexual minority individual’s risk for mental health problems by impairing their emotion regulation, interpersonal effectiveness, or cognitive processes. Existing research suggests that emotion regulation, shame, and depression help to explain some of the mental health disparities that sexual minority individuals experience (18–22). One risk factor that is especially relevant to suicidality is maladaptive emotion regulation, the “conscious and nonconscious strategies [people] use to increase, maintain, or decrease one or more components of an emotional response” (23). Emotion regulation deficits have been identified as a key transdiagnostic factor for a variety of mental health outcomes, including suicide (24). This is evident in that a large proportion of individuals seeking psychological help have difficulty managing emotional experiences (24, 25). Poor emotion regulation is frequently considered an underlying mechanism across several psychiatric diagnoses because it provides temporary relief yet prevents an individual from coping and problem-solving effectively. Several theorists have proposed that suicidal behavior, including suicide attempts, may be a maladaptive emotion regulation strategy that an individual uses to reduce intense negative emotion [e.g., (26–30)]. Existing treatments for suicidality target emotion regulation strategies as a proposed mechanism of change. Therefore, suicide attempts may reflect a manifestation of underlying emotion dysregulation.

Emotion dysregulation may be especially important in sexual minority individuals compared to heterosexuals for several reasons. First, sexual minority individuals learn from society that their same-gender feelings are invalid, resulting in mistrust of their emotional experiences (31). Second, they may also feel increased pressure to hide emotional experiences that are “too gay” or do not conform to their expected roles (31). Multiple studies have demonstrated that sexual minorities individuals who defy traditional gender roles are at particular risk for internalized stigma, victimization, and discrimination, as well as worse psychosocial adjustment (32–34). As a result of societal invalidation, sexual minority individuals may be more likely to experience mistrust of their emotion experiences as well as pressure to hide their internal experiences.

Various research studies have found an effect of invalidating environment on emotion regulation difficulties [e.g., (35)]. Some preliminary research has examined the role of emotion regulation difficulties in explaining the link between minority stress and mental health outcomes. These studies suggest that emotion regulation difficulties mediate the relationship between minority stress and depression and anxiety (18, 36, 37). However, no study to date has examined whether emotion regulation difficulties mediate the relationship between minority stress and suicidal behavior.

While the literature consistently demonstrates that sexual minority populations are at increased risk for suicidal behavior, it is limited in a number of ways. First, most studies examining suicidal behaviors in sexual minorities have focused on youth and young adults while potentially overlooking the experiences of other adults, who may have experienced varying degrees of cultural stigma. Second, few studies have empirically examined mediators of the relationship between sexual minority status and suicidal behavior, despite research highlighting the relevance of social support and emotion regulation as risk factors. More research is needed to elucidate the relationship between sexual minority status and suicide attempt. Third, most research on suicide risk in sexual minorities has only included individuals who actively identify as LGB. However, sexual minority status and identity include multiple distinct dimensions: sexual orientation, sexual attraction, and sexual behavior. Research demonstrates that individuals often report discordance between the three dimensions (38). For example, an individual may identify as heterosexual but still report same-gender attraction and/or sexual behavior. Therefore, sexual minorities who do not identify as sexual minorities are not often included in existing research.

To address these gaps, the present study examined sexual minority status, social support, emotion regulation, and suicide attempt in a large sample of adults from both the community and college settings. We explored the following questions: a) is sexual minority status related to increased likelihood of having attempted suicide? b) does degree of social support mediate the relation between sexual minority status and suicide attempt? c) does emotion regulation mediate the relation between sexual minority status and suicide attempt? and d) do the two mediators work in sequence? Hypotheses were as follows: 1) sexual minority status would be associated with greater likelihood of suicide attempt, 2) degree of social support would independently mediate the relation between sexual minority status and suicide attempt, 3) emotion regulation would independently mediate the relation between sexual minority status and suicide attempt, and 4) social support and emotion regulation, in sequence, would mediate the association between sexual minority status and suicide attempt.

## Methods

### Participants

Participants consisted of 388 adults recruited both in-person (*N* = 216) and online (*N* = 172) for a study examining self-injury and suicidality in sexual minorities. The in-person sample included adults who explicitly identified as heterosexual (*N* = 105) and adults who explicitly identified as a sexual minority (*N* = 111). All samples were combined in the present study due to similar methods and measures utilized, and to ensure that there was a wide range of responses available for comparison purposes between heterosexual and sexual minority participants.

Participants responded to announcements and messages specialized toward sexual minorities and individuals interested in participating in psychology tasks, and recruitment materials did not mention self-injury or suicidality. Participants were 18–64 years old (M = 25.41, SD = 9.36). Of the 388 participants, 78% (*n* = 303) were sexual minorities and 22% (*n* = 85) were exclusively heterosexual. Sexual minority individuals included those who endorsed non-heterosexual identity, attraction, and/or behavior. Of sexual minorities, 84% reported non-heterosexual sexual orientation (*n* = 256), 95% reported same-gender sexual attraction (*n* = 291), and 77% reported same-gender sexual behavior in their lifetime (*n* = 232). Ninety-one percent of the sample was cisgender (*n* = 356).Other demographics are reported in **Table 1**.

**Table 1:** Description of sample demographics (*N* = 388).

Variable	%	N
Sexual identity		
Bisexual/pansexual	38%	147
Heterosexual	33%	129
Gay/Lesbian	22%	84
Asexual	4%	17
Other	2%	8
Don’t know/do not wish to report	1%	3
Gender Identity		
Female	64%	247
Male	29%	112
Transfemale/woman	0.5%	2
Transmale/man	0.8%	3
Genderqueer/gender non-conforming	5.9%	23
Other	0.3%	1
Race		
White	51%	201
Asian	16%	64
Hispanic	12%	48
Black/African-American	11%	44
Native American	2%	7
Other/Multiracial	8%	21
Ethnicity		
Hispanic/Latinx	15%	60
Not Hispanic/Latinx	85%	328
Annual Household Income		
>$90,000	26%	102
$40,000–$89,999	34%	132
$20,000–$39,999	21%	83
<$19,000	18%	69

### Procedures

For the in-person subsample, sexual minority individuals were recruited through flyers and advertisements in the Piscataway, New Jersey area as well as through the Rutgers University Human Subjects Pool, and heterosexual participants were recruited through the Rutgers Humans Subjects Pool. The online study sample consisted exclusively of sexual minority participants recruited *via* Amazon Mechanical Turk (MTurk: www.mturk.com), an online venue where individuals can participate in online opportunities for nominal payments (39). Sample demographics from mTurk have been demonstrated to be at least as representative and diverse as conventional samples (Amazon.com) and use of mTurk allowed for the recruitment of older sexual minority individuals. This platform has previously been used as a valid method in the study of suicidality and other mental health issues [e.g., (40)], as well as in studying sexual minority individuals [e.g., (41)]. A prescreen questionnaire confirmed participant age and sexual orientation before completing any study procedures. To ensure data quality, attention checks were used in the survey (e.g., “If you are paying attention, select ‘1—Never’ for your answer”). Of 1,287 people who responded to the study’s post on MTurk, 742 completed the prescreening questionnaire and 249 were eligible. Of the 249 who were eligible, 214 completed consent. Of these 214 participants, 172 completed study procedures, 20 chose to stop participation, and 22 failed attention checks.

All participants completed a one-time 30-min battery of self-report questionnaires, which included a variety of mental health-related and LGBQ-related indices. All participants completed the same questions assessing sexual identity, sexual attraction, and sexual behavior. The study survey was hosted on Qualtrics, a HIPAA-compliant data collection platform. Data entered on Qualtrics were not tied to any identifying information. As compensation for study participation, in-person sexual minority participants received $15, in-person student participants received course credit, and online sexual minority participants received $1 through MTurk. Compensation amounts were commensurate with what is typically offered on the respective platforms. This study was approved by the Institutional Review Board at [University].

### Measures

#### Sexual Minority Status

Sexual orientation information was assessed using questions recommended in the literature (42–44). Three dimensions of sexual minority status were assessed: current sexual orientation identification (“How do you identify?”), current attraction (“Are you sexually attracted to or aroused by:”), and lifetime sexual behavior (“With whom have you had sexual experiences in your lifetime)?. Participants were designated as sexual minorities if they endorsed non-heterosexual identity, attraction, and/or behavior.

#### Social Support

The Multidimensional Scale of Perceived Social Support (MSPSS; 45) is a 12-item measure of perceived social support in a variety of social domains, including family (e.g., “My family really tries to help me”), friendships (e.g., “I can count on my friends when things go wrong”), and significant others (e.g., “I have a special person who is a real source of comfort to me.”). The MSPSS has demonstrated good test-retest reliability, validity, and internal reliability (46). The current study utilized the total score rather than specific subscales, which demonstrated good internal reliability in our sample (α = 0.90).

#### Emotion Regulation

The Difficulties with Emotion Regulation Scale (DERS) was used to assess problems with emotion regulation across six domains: nonacceptance of emotional responses, difficulties engaging in goal-directed behavior, impulse control difficulties, lack of emotion awareness, limited access to emotion regulation strategies, and lack of emotion clarity. Examples of items include “I know exactly how I am feeling,” and “When I am upset, I feel out of control.” The DERS has been shown to have good internal consistency, validity, and test-retest reliability (47). The DERS demonstrated excellent reliability in our sample (α = 0.96).

#### Suicide Attempt

Lifetime history of suicide attempt was assessed using a single item asking, “Have you ever in your life hurt yourself on purpose with the hope that you would die as a result?” Response options included “yes” or “no.” Research has demonstrated that individuals are as willing to disclose suicidal behavior on self-report as compared to in a clinical interview (48). Lifetime suicide attempt has been assessed using one item in past studies [e.g., (49)].

### Data Analysis

**Figure 1:**
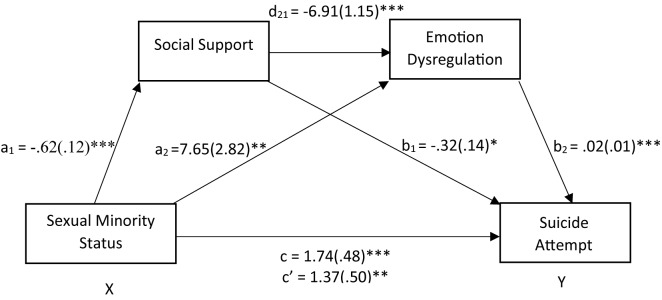
Social support and emotion dysregulation in sequence mediated the relationship between sexual minority status and suicide attempt. After adding the mediators, there is a significant indirect effect of sexual minority status on suicide attempt. The coefficients shown above are unstandardized. *Significant at the.05 level (2-tailed). **Significant at the.01 level (2-tailed). ***Significant at the.001 level (2-tailed).

**Figure 2:**
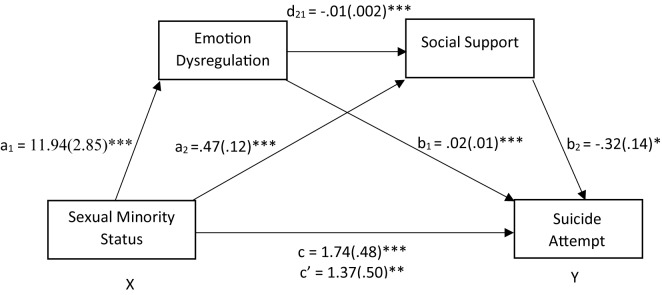
Emotion dysregulation and social support in sequence mediated the relationship between sexual minority status and suicide attempt. After adding the mediators, there is a significant indirect effect of sexual minority status on suicide attempt. The coefficients shown above are unstandardized. *Significant at the.05 level (2-tailed). **Significant at the.01 level (2-tailed). ***Significant at the.001 level (2-tailed).

First, we calculated correlations between all variables of interest to ensure that variables were related to one another in the expected directions. Correlations were corrected for multiple comparisons using the false discovery rate (FDR) correction to reduce the possibility of Type I errors (50). Next, we investigated whether social support and emotion regulation each independently mediated the effect of sexual minority status on lifetime suicide attempt. Serial mediation analyses were conducted as outlined by Preacher and Hayes (51) using SPSS Statistics 23 and the PROCESS macro. Specifically, we examined the total effect of sexual minority status on lifetime suicide attempt (c path in **Figures 1** and **2**), the relationship between sexual minority status (a paths), the effect of each mediator, social support or emotion regulation, on suicide attempt (b paths), the effect of social support on emotion regulation (d path), and the direct effect of sexual minority status on lifetime suicide attempt after adding the mediators to the model in sequence (c’ path). The indirect effect of sexual minority status on suicide attempt was tested using bootstrapping procedures, which make fewer assumptions about the sampling distribution. This procedure involves computing unstandardized indirect effects for each of 5,000 bootstrapped samples and calculating the 95% confidence interval (52). In order to examine the directionality of our effects, we also tested the reverse serial mediation model with emotion regulation as the first mediator and social support as the second. All analyses were re-run with gender and sampling method (online vs. in-person) as covariates to ensure that these variables were not driving effects. We also checked whether sampling method moderated effects.

## Results

### Preliminary Analyses

**Table 2** presents pairwise Pearson correlations (two-tailed), means, standard deviations, ranges, and normality estimates for the study variables (sexual minority status, social support, emotion regulation, and attempts). All correlations reported are false discovery rate—corrected for multiple comparisons. As shown in **Table 2**, sexual minority status was positively correlated with presence of suicide attempt and emotion dysregulation and negatively correlated with social support. Presence of suicide attempt was positively related to emotion dysregulation and inversely related to social support. Emotion dysregulation was inversely related to social support.

**Table 2:** Pearson’s and point-biserial correlations, means, standard deviations, ranges, and normality estimates for study variables.

Variable	1	2	3	4
1. Sexual minority status	—			
2. Presence of suicide attempt	.208**	—		
3. Social support	-.224**	-.222**	—	
4. Emotion dysregulation	.269**	.278**	-.331**	—
M *(SD)*	–	—	5.24 (1.16)	89.51 (27.02)
Range	0–1	—	1.33–7	37–155
Skewness *(SE)*	—	—	-.67 (.13)	.32 (.12)
Kurtosis *(SE)*	—	—	.19 (.25)	-.80 (.25)
Percentage that endorsed	78	13	—	—

*FDR-corrected correlation is significant at the 0.05 level 2-tailed).

**FDR-corrected correlation is significant at the 0.01 level (2-tailed).

### Serial Mediation Analyses

Serial mediation analyses are summarized in **Figures 1** and **2**. We first demonstrated that sexual minority status was associated with greater likelihood of previous suicide attempt (b = 1.74, SE =.48, OR = 5.71, p < .001). The odds of lifetime suicide attempt increased by 63% for sexual minority individuals. Results also indicated that sexual minority status was a significant predictor of social support [b = -.62, SE =.12, t(382) = -5.13, p < .001] and emotion dysregulation [b=7.65, SE=2.82, t(381)=2.82, p < .01]. The direct effect of social support as the first mediating variable on the second mediating variable of emotion regulation was at the significant level [b=-6.91, SE=1.15, t(381)=-6.00, p < .001]. A review of the direct effects of mediating variables on suicide attempt showed that the effects of social support (b= -.32, SE=.14, Z=-2.31, p=.02, OR=.73) and emotion dysregulation (b=.02, SE=.01, Z=3.92, p < .001; OR = 1.03) were significant. These findings indicated that the odds of a suicide attempt being reported decreased by approximately 27% per unit increase in social support and increased by approximately 3% per unit increase in emotion dysregulation. When sexual minority status and all mediating variables were simultaneously entered into the equation, the relation between sexual minority status and suicide attempt was still significant (b = 1.37, SE =.50, Z = 7.53, p < .01; OR = 3.92). With both mediators included in the model in sequence, the odds of lifetime suicide attempt almost tripled for sexual minority individuals. The indirect effects tested using a bootstrap estimation approach with 5,000 samples were significant for the first mediator [Social Support indirect b =.50, SE =.14, 95% confidence interval (CI) =.26,.82], second mediator [Emotion Regulation indirect b =.20, SE =.09, 95% confidence interval (CI) =.05,.39], and both mediators in sequence [b =.11, SE =.05, 95% confidence interval (CI) =.04,.20].

For the reverse mediation model, sexual minority status was a significant predictor of emotion dysregulation [b = 11.94, SE = 2.85, t(384) = 4.19, p < .001] and social support [b = -.47, SE =.12, t(384) = -3.99, p < .001]. The direct effect of emotion regulation on social support was at the significant level [b = -.01, SE =.002, t(384) = -6.00, p < .001]. The direct effects of the mediating variables, emotion dysregulation (b=.02, SE =.01, Z = 3.93, p < .001, OR = 1.03), and social support (b = -.32, SE =.14, Z = -2.31, p =.02; OR =.73), were both significant. Therefore, the odds of a suicide attempt being reported increased by approximately 3% per unit increase in emotion dysregulation and decreased by approximately 27% per unit increase in social support. When sexual minority status and all mediating variables were simultaneously entered into the equation, the relation between sexual minority status and suicide attempt remained significant (b = 1.37, SE =.50, Z = 2.74, p < .01; OR = 3.92). With both mediators included in the model in sequence, the odds of lifetime suicide attempt almost tripled for sexual minority individuals. The reverse mediation model with emotion regulation as the first mediator and social support as the second mediator was also significant [b =.05, SE =.03, 95% confidence interval (CI) =.004,.10].

To ensure the effects were not a function of gender and sampling, we ran the analyses with these variables with covariates. Findings were unchanged when covariates were entered. Furthermore, moderation analyses in which sampling was examined as a moderator of study independent variables demonstrated that sampling method did not moderate any effects.

## Discussion

The current study examined the relationship between sexual minority status, social support, emotion regulation, and history of suicide attempt in a community sample of adults. As hypothesized, sexual minority status was associated with greater likelihood of prior suicide attempt. Sexual minority status was also related to less social support and greater difficulties with emotion regulation. Social support was inversely related to prior suicide attempt, whereas emotion regulation difficulties were directly related to prior suicide attempt. Findings demonstrated that social support and emotion regulation, independently and in sequence, mediated the relationship between sexual minority status and suicide attempt. Contrary to our hypotheses, the reverse mediation model, with emotion regulation as the first mediator and social support as the second, was also significant. These results still held after controlling for gender and sampling method, and sampling method did not moderate effects.

These results demonstrate that social support and emotion regulation may play a role in explaining the relationship between sexual minority status and suicide attempt. This is consistent with previous research. Emotion regulation has been examined as a transdiagnostic factor for various psychopathologies (24) and has been shown to explain the relationship between minority stress and negative mental health outcomes (17). Similarly, interpersonal factors are implicated in both minority stress as well as in theories of development of suicide risk [e.g., (12, 16)]. Our findings support existing literature highlighting the relevance of these factors to both sexual minority status and suicide risk.

Findings from the serial mediation model suggest that social support and emotion regulation may contribute to the relationship between sexual minority status and suicide attempt. Emotion regulation mediated the relation between social support and suicide attempt, while social support mediated the relation between emotion regulation and suicide attempt. These findings suggest that effects between social support and emotion regulation are likely bidirectional. However, based on existing theory and research, poor social support likely leads to poor emotion regulation (17). Based on the psychological mediation framework, psychological risk factors explain the link between minority stress and adverse outcome. It is more likely that individuals first develop emotion regulation difficulties as a result of an unsupportive environment, rather than poor emotion regulation skills destroying the social support network. This study is consistent with the growing body of research suggesting that both minority stressors and psychological risk factors such as emotion dysregulation are relevant to negative mental health outcomes, such as suicide risk. Our findings extend the psychological mediation literature to the less studied outcome of suicide attempt. However, future research should explore trajectories of these various risk factors across time to better understand their interrelations.

The current study has several notable strengths. First, the use of a community-based in addition to convenience sample may be more generalizable than treatment-seeking samples. Second, this study included individuals who are sexual minorities on any of three dimensions of sexual minority status. Most studies typically assess presence or absence of sexual minority status based upon individual’s self-identity. Third, whereas there is a growing body of research on mediators of the relationship between sexual minority status and mental health outcome, few studies have utilized serial mediation models. This methodology allows for better understanding of how mediators are related. Lastly, this study reports on a dataset that has not been reported elsewhere.

It is also important to acknowledge several methodological limitations to the current study. The primary limitation of the study is the use of a cross-sectional sample. While this methodology allowed for a large, diverse sample, it prevents inferences about how variables are causally related as all data were collected at a single time point. It is possible that there were unobserved confounds. Future work would benefit from exploration of the causal relationship between sexual minority status, social support, emotion regulation, and suicide attempt by asking these research questions in a longitudinal design. Although our sample was well powered for simple group comparisons (e.g., minority status), our more advanced analyses, including serial mediation, likely require much higher levels of power (e.g., N > 1,000) to be adequately powered (52). However, because our effect sizes for multiple outcomes were so large, we believe that low power played less of a role in the outcomes of these analyses. Future studies should be sure to replicate the findings of this study with much larger samples, however.

Future work should also assess history of suicidal behavior in a more nuanced way, including examination of behaviors such as suicide gestures, aborted suicide attempts, and preparatory behavior in the absence of a suicide attempt, rather than with a single item indicating presence of past attempt. Additionally, while the current study conceptualized degree of social support as minority stressor, it may be more of a proxy or cause of other minority stressors such as victimization and discrimination. We acknowledge that degree of social support is only one facet of minority stress, and future studies should also examine other minority stressors, such as discrimination, microaggressions, among others. In addition to minority stressors, future work might also examine adherence to traditional gender roles and IPT factors. While this study did not have a large enough sample size to examine these variables in transgender individuals, this may be a worthwhile endeavor as this subgroup experiences unique stressors. Lastly, there has been growing attention in the area of resilience in the study of minority stress (53). Future work should consider resilience in addition to risk and stress.

If replicated in longitudinal design, these findings may have relevant clinical implications. The results suggest that lower emotion dysregulation and greater social support may function as a buffer against suicide attempt, particularly in sexual minority individuals. Therefore, clinicians may benefit from identifying sexual minority individuals with low social support and deficits in emotion regulation as a group at higher risk of suicide. In order to reduce individuals’ suicide risk and increase their general emotion regulation abilities, interventions may focus on increasing social support. Current treatments tailored toward sexual minority individuals, such as cognitive behavioral therapy adapted for sexual minorities, already incorporate interventions that target minority stress and emotion regulation processes [e.g., (54)]. Although longitudinal research is needed to better substantiate the interrelations between these variables, clinicians may want to consider a multi-faceted approach to treatment, which simultaneously addresses social support and emotion regulation processes as a critical point of suicide intervention in sexual minority individuals.

## Data Availability Statement

The datasets generated for this study will not be made publicly available. We specified in the consent forms that data would be presented in aggregate only. Requests to access these datasets should be directed to the corresponding author.

## Ethics Statement

The studies involving human participants were reviewed and approved by Rutgers University. The patients/participants provided their written informed consent to participate in this study.

## Author Contributions

CC conceived of the present study, participated in its design and coordination, conducted statistical analyses, and drafted the manuscript. KF devised the larger study from which this subset of data is drawn, collected the data, and helped to revise the manuscript. ES supervised the larger study from which the data originated, supported data collection, and helped to draft and revise the manuscript. All authors read and approved the final manuscript.

## Funding

The study was funded by The New Jersey Psychological Association.

## Conflict of Interest

The authors declare that the research was conducted in the absence of any commercial or financial relationships that could be construed as a potential conflict of interest.
